# Ocular biodistribution of bevasiranib following a single intravitreal injection to rabbit eyes

**Published:** 2008-05-28

**Authors:** Nadine S. Dejneka, Shanhong Wan, Ottrina S. Bond, Douglas J. Kornbrust, Samuel J. Reich

**Affiliations:** 1 OPKO Ophthalmics, Miami, FL; 2 Preclinsight, Reno, NV

## Abstract

**Purpose:**

The primary objective of these investigations was to determine the ocular biodistribution of bevasiranib, a small interfering RNA (siRNA) targeting vascular endothelial growth factor A (VEGF-A), following a single intravitreal injection to rabbit eyes.

**Methods:**

A tissue distribution and pharmacokinetic study was conducted with ^3^H-bevasiranib prepared in balanced-salt solution (BSS). Single doses of either 0.5 mg/eye or 2.0 mg/eye of ^3^H-bevasiranib were given by intravitreal injection to Dutch-Belted rabbits (both eyes were treated). Subgroups of rabbits were serially-sacrificed at various times up to 7 days following dosing for collection of tissue samples. The right eye of each rabbit was collected whole, and the left eye was dissected to isolate five ocular tissues. All samples were analyzed by liquid scintillation counting to determine the concentrations of bevasiranib equivalents. An ocular disposition study was also performed with non-radiolabeled bevasiranib, which was administered to Dutch-Belted rabbit eyes via intravitreal injection at a dose of 2.0 mg/eye. Twenty-four hours post-dose, the eyes were enucleated and dissected into eight individual ocular structures that were analyzed for intact bevasiranib using a locked nuleic acid (LNA) noncompetitive hybridization-ligation enzyme-linked immunosorbent assay.

**Results:**

Following intravitreal injection of 0.5 mg or 2.0 mg radiolabeled bevasiranib to Dutch-Belted rabbits, bevasiranib was detected in the vitreous, iris, retina, retinal pigment epithelium (RPE), and sclera (+choroid). As expected, the highest concentrations were found in the vitreous, and vitreous levels steadily decreased over time, while concentrations of radioactivity in the other ocular tissues increased to maximum values between 24 h and 72 h after dosing. Of these tissues, the highest concentration of radioactivity was detected in the retina. The LNA assay further confirmed the presence of intact bevasiranib in these tissues 24 h following intravitreal injection of non-radiolabeled bevasiranib (2 mg/eye).

**Conclusions:**

These studies demonstrate distribution of bevasiranib throughout the eye following intravitreal injection, including extensive uptake into the retina.

## Introduction

Age-related macular degeneration (AMD) is the leading cause of severe vision loss in the elderly [1,2]. Although the etiology of the disease is still not completely elucidated, it is clear that vascular endothelial growth factor (VEGF) plays a major role in the disease process. VEGF is a key mediator of pathological and physiologic angiogenesis and is increased in neovascular membranes of patients with AMD [3-6]. Pegaptanib, an RNA aptamer directed against VEGF-A, and ranibizumab, a monoclonal antibody fragment that binds VEGF-A, have both been approved by the United States Food and Drug Administration for the treatment of neovascular AMD [7,8].

RNA interference (RNAi) is a ubiquitous and endogenous gene silencing mechanism found in eukaryotic cells [9]. Its catalytic nature allows for one small interfering RNA (siRNA) to guide the cleavage of thousands of mRNAs, resulting in effective gene silencing [9]. Studies in mouse models of retinal and choroidal neovascularization have demonstrated the potential utility of this technology for the treatment of ocular disorders, and clinical trials are currently underway to test the ability of small interfering RNAs (siRNAs) targeting VEGF-A (bevasiranib) or VEGFR1 (AGN211745/SIRNA-027) to treat wet AMD [10,11]. Both siRNAs are being delivered to the eye via intravitreal injections.

This paper describes results from a study designed to assess the biodistribution of bevasiranib after an intravitreal injection in rabbits. To accomplish this, bevasiranib was radiolabeled with tritium (^3^H) and injected intravitreally into rabbit eyes once. Biodistribution and kinetics of radiolabeled bevasiranib post-injection were studied over a 7 day period. In addition, a bevasiranib-specific locked nucleic acid (LNA) assay was developed and validated. It was used to confirm the presence of intact bevasiranib in ocular tissue following a single intravitreal injection of the non-radiolabelled siRNA to rabbit eyes. Together these studies demonstrate the distribution of bevasiranib in the vitreous, retina, retinal pigment epithelium (RPE), sclera, and choroid of rabbits after a single intravitreal injection.

## Methods

All animal studies were performed in accordance with the ARVO Statement for the Use of Animals in Ophthalmic and Visual Research.

### siRNA

The sense strand sequence of bevasiranib is 5’-ACC UCA CCA AGG CCA GCA CdTdT-3’, while the antisense strand sequence is 5’-G UGC UGG CCU UGG UGA GGUdTdT-3’. ^3^H-Bevasiranib was synthesized by TriLink Bio Technologies, Inc. (San Diego, CA). On the day of dosing, the lyophilized powder was dissolved in the required volume of sterile balanced salt solution (BSS), pH 6.8–8.0, osmolality 334 mOsmol/l (Baxter Healthcare Corp, Deerfield, IL), with stirring. The test article formulation was prepared at a nominal concentration of 10 mg/ml (200 μCi/ml) or 40 mg/ml (160 μCi/ml). A correction factor of 1.11 was applied in dispensing the ^3^H-bevasiranib to adjust for purity. Both solutions were filtered through a 0.2 μm Gelman Acrodisc filter (Pall Life Sciences, Ann Arbor, MI) into sterile vials. The specific activity was 20 µCi/mg (for the low dose) and 4 µCi/mg (for the high dose).

Non-radiolabeled bevasiranib used for rabbit studies was obtained from Transgenomic (Omaha, NE). Bevasiranib was dissolved in 5% dextrose in water (D5W) forming a 40 mg/ml solution. The solution was filtered through a PALL Life Sciences Acrodisc® 13 mm syringe filter with a 0.2 μm HT Tuffryn® Membrane (Pall Life Sciences). For intravitreal injection, filtered material was loaded into 1 ml tuberculin syringes fitted with 30 gauge needles and stored at 4 °C until it was administered.

### In vivo ^3^H-bevasiranib studies

A total of 16 male and 16 female Dutch-Belted rabbits were used. At the onset of treatment, the animals were approximately 5 months in age, and their body weights ranged from 1.9 to 2.9 kg.

Animals were randomized into 2 study groups: Group 1, 0.5 mg ^3^H-bevasiranib/eye; Group 2, 2.0 mg ^3^H-bevasiranib /eye. Immediately before anesthesia, mydriatic drops (1% Mydriacyl) were applied to the eyes. Prior to dosing, the animals received an intramuscular sedative injection of a ketamine and xylazine cocktail. The conjunctivas were then flushed with a 1:10,000 solution (equivalent to 0.1 mg/ml) of benzalkonium chloride 50% NF (Spectrum Lab Products, Inc., New Brunswick, NJ) prepared in 0.9% (w/v) sodium chloride for injection USP (Baxter). A local anesthetic (Alcain, 0.5%) was applied to each eye. For each injection, a new insulin syringe (with pre-fitted needle) was used. Dose formulation was administered by intravitreal injection in both eyes at a dose volume of 50 µl/eye, using a binocular indirect ophthalmoscope to confirm needle placement. The ophthalmologist examined the eyes immediately following treatment (indirect ophthalmoscopy and slit-lamp biomicroscopy) and documented any abnormalities caused by the dosing procedure. Following examination, gentamycin ophthalmic drops were applied to each eye and an ocular lubricant (Tears Naturale®, Alcon, Fort Worth, TX) was used if considered appropriate by the ophthalmologist.

Animals were euthanized at predetermined times (6, 24, 72, and 168 h post-injection) by intravenous injection of Euthanyl® (Bimeda-MTC Animal Health Inc., Cambridge, Ontario, Canada; approximately 200 mg/kg). Two female animals and two male animals were sacrificed at each time point. Following euthanasia, tissue was collected. The right eye was removed intact. The left eye was dissected to collect aqueous fluid, iris, vitreous fluid, retina and sclera (including choroid). All samples were stored at -80 °C.

### Radioactivity measurements

The total weights of the tissue samples were recorded. Tissue samples were solubilized in 35% tetraethylammonium hydroxide (TEAH). The solubilized samples, or duplicate aliquots thereof, were then mixed with liquid scintillation fluid before radioactivity measurements.

Radioactivity measurements were conducted by liquid scintillation spectroscopy. Each sample was counted for 5 min or to a two-sigma error of 0.1%, whichever occurred first. All counts were converted to absolute radioactivity (dpm) by automatic quench correction based on the shift of the spectrum for the external standard. Samples that exhibited radioactivity less than or equal to twice the background values were considered as zero for all subsequent manipulations.

All radioactivity measurements were entered into a standard computer database program (Debra Version 5.2) for the calculation of concentrations of radioactivity (dpm/g and mass eq/g) and percentage of administered radioactivity in each sample. Tissue concentrations of radioactivity were calculated initially in dpm/g, and then mass eq/g (assuming intact siRNA) was calculated on the basis of the measured specific activity (dpm/mg or appropriate mass unit) of radiolabeled test article in the dose solution. Total tissue content was calculated based on the total tissue weight.

Non-compartmental pharmacokinetic parameters were estimated for the ocular tissue data, using SAS Version 8.1, and included area under the concentration versus time curve (AUC), terminal half-life (t1/2el), terminal rate constant (kel), the highest concentration observed (Cmax), and time at which the highest concentration occurred (tmax). The Cmax was obtained by data inspection. The AUC was calculated by application of the trapezoidal rule and kel was obtained by linear regression analysis of selected time points in the terminal phase of the concentration versus time curves [12]. The apparent terminal half-life (t1/2el) was calculated as follows: t1/2el=ln2/kel. For all time deviations greater than 10%, the actual time collection was used for estimation of the parameters.

### In vivo non-radiolabeled bevasiranib studies

Male Dutch-Belted rabbits, ranging in weight from 2.0 to 2.4 kg, were used. Animals were anesthetized with a ketamine/xylazine (8 ml ketamine: 1 ml xylazine) cocktail at 0.5 ml/kg on Day 1. Eyes were then dilated with 2–3 drops of 1% Tropicacyl® (tropicamide; Alcon) followed by 2-3 drops of 0.5% proparacaine as an analgesic. Two rabbits received bilateral intravitreal injections of 5% dextrose for injection (D5W). An additional three rabbits received intravitreal injections of bevasiranib prepared in D5W (Note: 5 of 6 eyes were treated.). All injections were 50 μl in volume and made with 1 ml syringes fitted with 30 gauge needles. Rabbits dosed with bevasiranib received 2 mg/eye. The following day (24 h post-dosing), animals were euthanized. Both eyes were collected from each animal, and the eyes were dissected to obtain 8 tissues/eye. Tissues were frozen on dry ice and ethanol and subsequently stored at -80 °C until further processing.

Homogenates were prepared from each tissue sample. Briefly, sterile phosphate-buffered saline (PBS) was added to each sample (500 μl-2 ml), and tissue was sonicated with a Branson Sonifier 150D (Branson, Danbury, CT) until the tissue was completely disrupted.

Following tissue disruption, samples underwent a series of 3 freeze/thaw cycles to further lyse the cell membranes. Each cycle consisted of freezing the samples in a dry ice/ethanol bath, thawing the samples in a 37 °C water bath and vortexing the samples. Samples were subsequently centrifuged in a microfuge at ~18,000x g for 10 min. Supernatants were transferred to fresh tubes, and all samples were stored at -80 °C. Protein concentrations were determined on the supernatant samples using the BCA^TM^ Protein Assay Kit (Pierce Chemical, Rockford, IL), and the samples were subsequently used for LNA analysis.

### Reagents and chemicals for locked nuleic acid analysis

A template probe, template probe working solution, wash buffer, ligation probe, ligation probe working solution, anti-digoxigenin-AP working solution, and detection solution were prepared. The template probe was 5’-GAA TAG CGA A+A AC+ CTC +AC C+A AG+ GCC +AG C+A C-(BIOTIN)-3’ (capital letters=DNA; +=LNA; (BIOTIN)= biotin-TEG; Sigma, St. Louis, MO). It was rehydrated in 1X PBS to a final concentration of 10 μM. The template probe working solution was prepared by combining 150 μl of 10 μM template probe with 30 ml of 6X SSPE/0.24% Tween-20 to obtain a final concentration of 50 nM template probe. The wash buffer was 1X Tris-buffered saline (TBS), 0.1% Tween-20. The ligation probe was 5’-(PHOSPHATE)-TCG CTA TTC-(DIGOXIGENIN)-3’ (Invitrogen, Carlsbad, CA). The probe was rehydrated in 1X One-Phor-All Buffer PLUS (10X One-Phor-All Buffer Plus; Amersham Biosciences Corporation, Piscataway, NJ) to a final concentration of 10 μM. The ligation probe working solution was prepared by combining 10X One-Phor-All Buffer Plus with T4 DNA ligase, ATP, water and 10 μM ligation probe. The final concentration of the solution was 75 nM ligation probe, 2 units/ml T4 DNA ligase, 0.10 mM ATP, 1X One-Phor-All Buffer Plus. The Anti-digoxigenin-AP working solution was prepared by combining 11 μl of anti-digoxigenin-AP, Fab fragments (Roche Applied Sciences, Indianapolis, IN) with 22 ml of Super-Block buffer working solution. Super-Block buffer working solution was prepared by adding 10 ml of SuperBlock® Blocking Buffer in TBS (Pierce Chemical) to 90 ml of ultra pure water. Detection solution was prepared by combining two, 5 mg PNPP (p-nitrophenyl phosphate disodium salt) tablets (Pierce Chemical) with 8 ml water and 2 ml of 5X diethanolamine substrate buffer concentrate (Pierce Chemical).

### Locked nucleic acid enzyme-linked immunosorbent assay protocol

The protocol was adopted from a previously described method and optimized for bevasiranib [13]. Bevasiranib standard siRNA and protein samples (0.5–40 μg) were aliquoted into 1.5 ml Eppendorf tubes. The final volume was adjusted to 150 μl with PBS. PBS served as a negative control. 300 μl of template probe working solution was added to each tube, and the contents were vortexed. Samples were incubated at 90 °C for 10 min, cooled at room temperature for 15 min, placed in an ice water bath for 5 min and refrigerated at 4 °C for 1 h. Tubes were subsequently centrifuged at 16,000x g for 15 min at 4 °C. Supernatant (200 μl/well; duplicate wells) was then transferred to Reacti-Bind^TM^ NeutrAvidin^TM^ Coated High Binding Capacity (HBC) Clear 96 well plates (Pierce Chemical). The plate was covered with an adhesive strip and placed in a 37 °C incubator for 30 min. The plate was then refrigerated at 4 °C for 10 min and washed 4 times with wash buffer. Care was taken after the final wash to remove all traces of wash buffer. Similar care was taken for all subsequent washes that followed.

Ligation probe working solution (200 μl) was subsequently added to each well. The plate was covered with an adhesive strip and incubated at room temperature on an orbital shaker (100 rpm) for 1 h. The plate was then washed twice with wash buffer, 3 times with ultra pure water (wells were soaked for 3 min during each water wash), and then one last time with wash buffer.

Anti-digoxigenin-AP working solution (200 μl) was added to each well. The plate was covered with an adhesive strip and incubated on an orbital shaker (100 rpm) for 30 min at room temperature. The plate was then washed 5 times with wash buffer.

Then 100 μl of detection solution was added to each well. The plate was covered with aluminum foil to maintain dark conditions and was incubated at room temperature for 30 min. NaOH (2 N; 50 μl) was added to each well. The optical density of each well was determined at 405 nm using a microplate reader.

### Locked nucleic acid enzyme-linked immunosorbent assay validation studies

Prior to tissue analysis, several validation procedures were completed. Linearity of dilution of bevasiranib in PBS was determined in the absence of protein and intra-assay precision and accuracy were determined. The lower limit of quantification (LLOQ) was 6.25 ng/ml, and the upper limit of quantification (ULOQ) was 200 ng/ml. Finally, selectivity of bevasiranib was determined in the presence of protein. Rabbit eye homogenates, devoid of bevasiranib, were used for these assays. Increasing concentrations of rabbit ocular tissue protein (0–75 μg/ml) had no effect on bevasiranib levels (Data not shown).

## Results

### Ocular responses to ^3^H-bevasiranib following a single intravitreal injection

No systemic treatment-related clinical signs were observed in the rabbits following a single intravitreal dose of ^3^H-bevasiranib.

Ophthalmological evaluations were performed pre-dose, directly after administration and at approximately 48 h and 144 h post-dose. For Group 1 (0.5 mg ^3^H-bevasiranib/eye) at 48 h, the most frequent observation was slight conjunctival hyperemia (5/16 eyes). In addition, small numbers of vitreous cells were observed in a single eye. By 144 h, no treatment related signs were observed in the eyes of the remaining animals in Group 1.

At 48 h after dosing, the most frequent observation for Group 2 animals (2.0 mg ^3^H-bevasiranib/eye) was congestion of the retinal vessels (11/16 eyes) followed by mild anterior uveitis (7/16 yes) and slight to moderate conjunctival hyperemia (7/16 eyes). The most severely affected animal also exhibited bilateral focal retinal hemorrhages. By 144 h, congestion of the retinal vessels was no longer present, but variable numbers of vitreous cells were observed in all of the eyes of the surviving animals. Focal opacity of the vitreous (6/8 eyes), slight aqueous flare (4/8 eyes) and focal vitreous hemorrhage (2/8 eyes) were also observed. In the absence of a placebo group, it is difficult to assign causality of the above mentioned, transient adverse events. Such events are commonly associated with injection procedures and manipulation of the ocular surface.

### ^3^H-bevasiranib localizes to the retina following a single intravitreal injection

Radioactivity derived from the ^3^H-bevasiranib was detected in all the ocular structures examined, and the values for percentage of the dose found in the tissues were calculated using the doses administered to the individual eye.

For males in Group 1, the highest mean concentration of radioactivity in the ocular tissues/fluids was 429 μg-eq/g, observed in the vitreous fluid of the left eye at 6 h after dosing (the first time point measured; Table 1). Thereafter concentrations in vitreous fluid decreased steadily over time to 118 μg-eq/g by 72 h post-injection and to 10.4 μg eq/g by 168 h post-dose (2.4% of the maximum value). Mean concentrations of radioactivity in the other ocular tissues from the left eye (retina, iris, sclera/choroid and aqueous fluid) increased from 6 h to maximum values at 24 or 72 h after dosing. Of these tissues, the highest concentration of radioactivity was observed in the retina at 72 h post-dose (mean 370 μg-eq/g), and the level of radioactivity in the retina did not diminish substantially between 72 and 168 h post-dose. Uptake into the aqueous fluid of the left eye was relatively low, with a mean concentration of 20 μg-eq/g at 24 h post-injection, diminishing to approximately 1 μg-eq/g by 168 h. Mean radioactivity concentrations for the whole right eye showed a similar pattern to that of vitreous fluid, with the mean Cmax at 6 h post-dose (205 μg-eq/g), decreasing to 18.3 μg-eq/g by 168 h.

**Table 1:** Mean concentration of radioactivity in ocular tissues of Dutch-Belted rabbits following a single intravitreal bolus injection of ^3^H-bevasiranib.

			Concentration of radioactivity, μg eq/g^a^			
**Group**	**Sex**	**Sample**	**6 h**	**24 h**	**72 h**	**168 h**
1	Males	Aqueous fluid	12.3	20.1	11	1.1
1	Males	Eye (right)	205	105.1	71.3	18.3
1	Males	Iris	12.8	31.7	50	21
1	Males	Retina	103.7	221.5	370.2	250.6
1	Males	Sclera (+ choroid)	65.4	88.7	54.3	27
1	Males	Vitreous fluid	429.3	273.9	118.6	10.4
1	Females	Aqueous fluid	3.9	28.9	10.5	1.2
1	Females	Eye (right)	175	162	59.4	21.1
1	Females	Iris	6.4	37.3	36.4	28.2
1	Females	Retina	91	193.8	342.8	278.3
1	Females	Sclera (+ choroid)	57	75.6	54.6	24.4
1	Females	Vitreous fluid	355.2	307.6	113.7	12.2
2	Males	Aqueous fluid	79.8	125.1	90.8	20.2
2	Males	Eye (right)	781.2	599.8	483	148.9
2	Males	Iris	79.3	134.9	188.6	126.7
2	Males	Retina	380.2	416	654.5	682.7
2	Males	Sclera (+ choroid)	340.6	329	207.7	100.6
2	Males	Vitreous fluid	1919.3	1357.1	777.8	200.7
2	Females	Aqueous fluid	119.9	126.9	52.1	19.9
2	Females	Eye (right)	818.2	553.9	438.6	159.3
2	Females	Iris	139.3	149	92.1	118.7
2	Females	Retina	436.1	439.9	449	628.1
2	Females	Sclera (+ choroid)	381.1	363	124.7	85.7
2	Females	Vitreous fluid	1748.3	1362.6	504.4	205.9

Eight male and eight female rabbits (total 16 rabbits) were used for each treatment group. Group 1 animals received 0.5 mg ^3^H-bevasiranib/eye via intravitreal injection. Group 2 animals received 2.0 mg/bevasiranib/eye. Two animals of each sex (total 4 rabbits) were sacrificed at predetermined time points (6, 24, 72, and 168 h). Whole eyes (right eyes) and dissected ocular tissues from left eyes were analyzed for levels of radioactivity. ^a^Mean, n=2

Table 2 presents the mean percentage of the administered dose found in each ocular structure. For males from Group 1, at 6 h after dosing, a mean of 92.1% of the administered dose remained in the right eye. At this time, the majority of the radiolabeled material (mean 83.1%) was found in the vitreous fluid (left eye data) with a further 5.5% found in the other tissues of the left eye. Mean amounts of radioactivity in whole eye, vitreous fluid and aqueous fluid generally decreased at later time points, while those in iris, retina and sclera generally increased, at least for the earlier time points. Similar trends were also observed in female animals receiving the 0.5 mg/eye treatment.

**Table 2:** Mean radioactivity content in tissue of Dutch-Belted rabbits following a single intravitreal bolus of ^3^H-bevasiranib.

			Percent of dose**^a^**			
**Group**	**Sex**	**Sample**	**6 h**	**24 h**	**72 h**	**168 h**
1	Males	Aqueous fluid	0.5	0.4	0.5	0.05
1	Males	Eye (right)	92.1	46.5	36.9	8.8
1	Males	Iris	0.1	0.2	0.3	0.1
1	Males	Retina	0.5	0.9	2	2.3
1	Males	Sclera (+ choroid)	4.4	6	3.8	1.7
1	Males	Vitreous fluid	83.1	41.2	20	1.7
1	Females	Aqueous fluid	0.2	1.4	0.5	0.1
1	Females	Eye (right)	85.7	76.1	29.6	10
1	Females	Iris	0.04	0.2	0.2	0.2
1	Females	Retina	0.5	1.2	2.3	2.8
1	Females	Sclera (+ choroid)	4.5	4.8	4	1.3
1	Females	Vitreous fluid	82.6	57.1	18.6	2
2	Males	Aqueous fluid	0.9	1.2	0.9	0.2
2	Males	Eye (right)	91.9	73.2	51.7	15.8
2	Males	Iris	0.1	0.2	0.3	0.2
2	Males	Retina	0.3	0.8	0.9	1.4
2	Males	Sclera (+ choroid)	7.5	6.4	3.4	1.3
2	Males	Vitreous fluid	65.1	54	37.5	8.1
2	Females	Aqueous fluid	1.2	1.1	0.4	0.2
2	Females	Eye (right)	90.5	60.1	47.7	17.5
2	Females	Iris	0.2	0.2	0.1	0.2
2	Females	Retina	0.5	0.7	0.9	1.5
2	Females	Sclera (+ choroid)	6.6	5.4	1.7	1.1
2	Females	Vitreous fluid	63.2	50.1	25.1	10.3

Male (n=8) and female (n=8) rabbits were treated with ^3^H-bevasiranib via intravitreal injection. Group 1 animals received 0.5 mg bevasiranib/eye. Group 2 animals received 2.0 mg/bevasiranib/eye. Two animals of each sex (total 4 rabbits) were sacrificed at predetermined times (6, 24, 72, and 168 h) post-injection. Whole eyes (right eyes) and dissected ocular tissues from left eyes were analyzed for levels of radioactivity. The mean percentage of radioactivity found in each ocular structure is reported. ^a^Mean, n=2.

When 2.0 mg ^3^H-bevasiranib was administered to rabbits (Group 2), males showed the highest mean concentration of radioactivity in the vitreous fluid of the left eye at 6 h post-dose (1,919 μg-eq/g; Table 1). Thereafter, concentrations in vitreous decreased to 201 μg-eq/g at 168 h post-dose (10.5% of the Cmax value). Mean concentrations of radioactivity in sclera were also maximal at 6 h (341 μg-eq/g), whereas those of the other ocular tissues from the left eye (retina, iris, and aqueous fluid) increased to maximum values at 24 (aqueous fluid), 72 (iris), or 168 h after dosing (retina). Of these tissues, the highest concentration of radioactivity was observed in the retina (mean 683 μg-eq/g); there was little change in the radioactivity level of the retina of Group 2 animals between 72 and 168 h post-dose. The mean radioactivity concentration for the intact right eye exhibited a similar pattern to that of vitreous, with the mean Cmax at 6 h post-dose (781 μg-eq/g), decreasing to 149 μg-eq/g by 168 h.

At 6 h after dosing, a mean of 91.9% of the administered dose remained in the right eye, with the majority of the radiolabeled material (mean 65.1%) found in the vitreous (left eye data) and 8.8% found in the other left eye tissues (Table 2). Mean amounts of radioactivity in whole eye, vitreous fluid and sclera decreased at later time points, while those in iris, retina and aqueous fluid generally increased, at least for the earlier time points. Once again, female rabbits showed similar results to that of the male counterparts in Group 2.

### Pharmacokinetics of radioactivity in the eye

Mean radioactivity concentration versus time data were used to calculate pharmacokinetic parameters for ocular tissue. AUC(0-t) values were calculated, where t corresponded to 168 h post-dose (the last time point taken). Female and male rabbits showed similar results (Table 3).

**Table 3:** Disposition kinetics of the total radioactivity in ocular tissues of Dutch-Belted rabbits following a single intravitreal bolus injection of ^3^H-bevasiranib.

			**t_max_**	**C_max_**	**t_last_**	**AUC_0-tlast_**	**AUC_0-inf._**	**%Extrapolation**	**k_el_**	**t_1/2el_**
**Group**	**Sex**	**Sample**	**(h)**	**(μg eq/g)**	**(h)**	**(μg eq·h/g)**	**(μg eq·h/g)**	**AUC_0-inf._**	**(h^-1^)**	**(h)**
1	Males	Aqueous Fluid	24	20.1	168	1654	a	A	a	a
1	Males	Eye (Right)	6	205	168	11936	13402	10.9	0.01	55.7
1	Males	Iris	72	50.1	168	5814	a	A	a	a
1	Males	Retina	72	370	168	47235	a	A	a	a
1	Males	Sclera (+ choroid)	24	88.7	168	8916	a	A	a	a
1	Males	Vitreous fluid	0	499	168	24725	25176	1.8	0.02	30
1	Females	Aqueous Fluid	24	28.9	168	1812	a	A	a	a
1	Females	Eye (Right)	6	175	168	12737	14281	10.8	0.01	50.7
1	Females	Iris	24	37.3	168	5282	a	A	a	a
1	Females	Retina	72	343	168	45527	a	A	a	a
1	Females	Sclera (+ choroid)	24	75.6	168	8280	a	A	a	a
1	Females	Vitreous fluid	0	372	168	24301	24840	2.2	0.02	30.7
2	Males	Aqueous Fluid	24	125	168	12589	a	A	a	a
2	Males	Eye (Right)	6	781	168	71089	85914	17.3	0.01	69
2	Males	Iris	72	189	168	25065	a	A	a	a
2	Males	Retina	168	683	168	98185	a	A	a	a
2	Males	Sclera (+ choroid)	6	341	168	34724	a	A	a	a
2	Males	Vitreous fluid	0	2154	168	139913	154893	9.7	0.01	51.7
2	Females	Aqueous Fluid	24	127	168	10334	a	A	a	a
2	Females	Eye (Right)	6	818	168	67323	85177	21	0.01	77.7
2	Females	Iris	24	149	168	18915	a	A	a	a
2	Females	Retina	168	628	168	82229	a	A	a	a
2	Females	Sclera (+ choroid)	6	381	168	29642	a	A	a	a
2	Females	Vitreous fluid	0	1896	168	117832	134198	12.2	0.01	55.1

^a^It was not possible to estimate the k_el_ with an acceptable degree of confidence due to an inability to characterize the terminal phase. Consequently, all parameters derived from this, t_1/2_, AUC_0-inf._ and % extrapolation AUC_0-inf._ were not estimated.

For male rabbits in Group 1, AUC(0–168h) values for retina, vitreous, whole right eye, sclera, iris and aqueous fluid were 47,235 μg-eq·h/g, 24,725 μg-eq·h/g, 11,936 μg-eq·h/g, 8,916 μg-eq·h/g, 5,814 μg-eq·h/g, and 1,654 μg-eq·h/g, respectively.

For whole right eye and vitreous, sufficient data were available to permit estimation of kel, the half-life of elimination, t1/2 and the AUC(0-inf). For the right eye, kel was 0.0124 h-1, t1/2 was estimated as 55.7 h, and the AUC (0-inf) was 13,402 μg-eq·h/g (% extrapolation (t-inf) 10.9%), whereas for vitreous fluid from the left eye, the same parameters were calculated as 0.02 h-1, 30.0 h and 25,176 μg-eq·h/g (% extrapolation [t-inf] 1.8%).

In contrast to the 0.5 mg/eye dose, male rabbits receiving 2.0 mg/eye (Group 2) achieved the highest AUC (0–168 h) value in the vitreous fluid (139,913 μg-eq·h/g). This was followed by retina (98,185 μg-eq·h/g), whole right eye (71,089 μg-eq·h/g), sclera (34,724 μg-eq·h/g), iris (25,065 μg-eq·h/g), and aqueous fluid (12,589 μg-eq·h/g).

For the right eye, kel, was 0.01 h-1, t1/2 was estimated as 69.0 h, and the AUC(0-inf) was 85,914 μg-eq·h/g (extrapolation [t-inf] 17.3%). For vitreous fluid from the left eye, the same parameters were calculated as 0.01 h-1, 51.7 h and 154,893 μg-eq·h/g (extrapolation [t-inf] 9.7%).

### Biodistribution of intact bevasiranib 24 h after intravitreal injection

The data in Table 4 are representative of a single experiment using 5 rabbits. The study was repeated on 3 additional occasions with similar results.

**Table 4:** Levels of intact bevasiranib in individual rabbit tissues following intravitreal injection.

		Total bevasiranib present in tissue sample (ngs) 24 h following intravitreal injection							
Animal ID	Treatment	Aqueous fluid	Vitreous fluid	Lens	Iris	Ciliary Body	RPE	Choroid	Retina
A1/OS	D5W	<LLOQ	<LLOQ	<LLOQ	<LLOQ	NA	<LLOQ	<LLOQ	<LLOQ
A1/OD	D5W	<LLOQ	<LLOQ	<LLOQ	<LLOQ	<LLOQ	<LLOQ	<LLOQ	<LLOQ
B1/OS	D5W	<LLOQ	<LLOQ	<LLOQ	<LLOQ	NA	<LLOQ	<LLOQ	<LLOQ
B1/OD	D5W	<LLOQ	<LLOQ	<LLOQ	<LLOQ	NA	<LLOQ	<LLOQ	<LLOQ
A2/OS	Bevasiranib	325.6 (0.02%)	>ULOQ	<LLOQ	18,358.3 (0.92%)	176.3 (0.01%)	806.8 (0.04%)	214.2 (0.01%)	236.5 (0.01%)
A2/OD	Bevasiranib	2,170.5 (0.11%)	>ULOQ	<LLOQ	1,572.3 (0.08%)	12.7 (0.001%)	422.6 (0.02%)	58.2 (0.003%)	2,335.1 (0.12%)
B2/OS	Bevasiranib	3,547.0 (0.18%)	>ULOQ	<LLOQ	18,943.8 (0.95%)	86.2 (0.004%)	645.3 (0.03%)	382.1 (0.02%)	410.8 (0.02%)
B2/OD	Bevasiranib	4,043.0 (0.20%)	>ULOQ	16,160.6 (0.81%)	27,534.9 (1.38%)	553.8 (0.03%)	>ULOQ	1,298.5 (0.06%)	749.8 (0.04%)
E5/OD	Bevasiranib	1,688.9 (0.08%)	114,171.9 (5.71%)	<LLOQ	2,371.0 (0.12%)	6,396.6 (0.32%)	2,695.6 (0.13%)	2,139.9 (0.11%)	1,690.2 (0.08%)

Rabbit eyes were treated with 50 μl of D5W or 2 mg bevasiranib, prepared in 50 μl D5W. Levels of bevasiranib were measured in individual rabbit tissues 24 h post-dosing using LNA analysis. The total bevasiranib in tissue is presented in ngs. Percentages in parentheses reflect the percent of total dose recovered from each tissue. Abbreviations: LLOQ means lower limits of quantification; ULOQ means upper limits of quantification; NA means not analyzed.

As anticipated, LNA analysis confirmed bevasiranib was not detected in any ocular tissue following intravitreal injection of the D5W vehicle. Twenty-four hours following intravitreal injection of 2 mg bevasiranib/eye, intact bevasiranib was not detected in lens (4/5 eyes). Bevasiranib was detected in aqueous fluid, vitreous fluid, iris, ciliary body, RPE, choroid and retina, with the highest level in the vitreous fluid, where the concentrations in 4/5 eyes were above the ULOQ.

## Discussion

The studies described herein have characterized the biodistribution of bevasiranib following a single intravitreal injection in Dutch-Belted rabbits. Bevasiranib is a double-stranded RNA oligonucleotide of 21 nucleotides in length having one strand with a primary sequence that has Watson–Crick homology to 21 nucleotides in the VEGF-A mRNA. Reich and colleagues previously demonstrated that bevasiranib decreased levels of hVEGF (imparted via an adenovirus vector) in mouse RPE cells in vivo following subretinal injection of bevasiranib [10]. The effect was specific, as an siRNA targeting EGFP had no effect. In addition, they demonstrated that an siRNA targeting murine VEGF was able to inhibit choroidal neovascularization (CNV) in a laser-induced model of CNV, while, a control siRNA targeting EGFP had no effect.

As a follow-up, we sought to determine the biodistriubtion of bevasiranib following intravitreal injection. The intravitreal route of administration was considered the likely route of delivery in treating VEGF-A mediated ocular diseases in man, such as neovascular-AMD and diabetic retinopathy. At 6 h post-dose (the first time point), the highest concentration of radioactivity in the tissues of all animals was found in the vitreous of the eye (i.e., the site of administration). Concentrations were also appreciable in the other ocular tissues investigated (aqueous fluid, iris, retina, and sclera). For these ocular tissues, the radioactivity generally (but not always) increased with time after dosing, indicating the migration of bevasiranib from the vitreous compartment into various tissues of the eye. Among the ocular tissues examined, the highest concentrations were present in the retina. The slow increase in radioactivity in the retina indicated that this radiolabeled material was transferred into and retained in the retina and was not the product of cross-contamination from the vitreous fluid. Radioactivity levels in retina generally did not decline substantially between Cmax and the last time point measured, indicating persistence of ^3^H-bevasiranib-related material in this target tissue for an extended period. The pattern of ocular distribution derived from ^3^H-bevasiranib was similar for both male and female rabbits at both dose levels.

Pharmacokinetic analyses of the radioactivity concentration versus time profiles for animals in Group 1 (0.5 mg/eye) indicated that the AUC (0–168 h) value for retina was greater than that of vitreous fluid, therefore supporting the interpretation that radiolabeled material had moved from the vitreous fluid and accumulated in the retina. For the Group 2 animals (2 mg/eye), this relationship was reversed although the mean AUC values for retina were again relatively high. This may reflect partial saturation of the rate of uptake of bevasiranib into the retina at the higher dose level. The pharmacokinetic parameters estimated for animals of both sexes in each dose group were similar, again indicating that there were no sex-related differences in the exposure of the animals to ^3^H-bevasiranib and/or its metabolites. The half-life of radioactivity in the vitreous fluid of both male and female rabbits following administration of the 0.5 mg/eye dose exceeded 24 h. In the animals that received the 2.0 mg/eye dose, the half-life of radioactivity in the vitreous fluid exceeded 48 h.

To confirm the presence of intact bevasiranib in the different ocular structures, we employed an LNA-based bioanalytical method that is specific for the intact siRNA (i.e., for one of the duplex strands). Since both strands of bevasiranib are present in equivalent amounts in the hybridization duplex, the validated hybridization method allows for the quantification of a single strand of bevasiranib only (i.e., the antisense strand), which is directly proportional to the concentration of the duplex bevasiranib (on a molar basis).

Aqueous fluid, vitreous fluid, lens, iris, ciliary body, RPE, choroid, and retina were assayed for intact bevasiranib 24 h post-injection. Bevasiranib was generally detected in all the ocular structures, the highest concentrations were consistently observed in vitreous fluid. The variation observed is suggestive of differences in bevasiranib distribution within the eye between eyes and between animals.

To treat neovascular AMD, bevasiranib must reach the RPE-Bruch’s membrane-choroidal complex. The radiolabeled bevasiranib studies show that it can reach the retina and the RPE-Bruch’s membrane choroidal complex. However, the measurement of total radioactivity leaves uncertainty about how much of the material detected in tissues reflect intact bevasiranib. Distribution of bevasiranib into the tissues would be expected to lead to its metabolism by tissue nucleases, however this would not be expected to occur immediately or completely. Indeed, LNA analysis confirmed the presence of intact bevasiranib in ocular tissues 24 h after intravitreal injection, suggesting bevasiranib is not only taken up by the tissues, but that some remains intact for an extended period of time. The levels of bevasiranib measured in the RPE, a tissue of interest in neovascular-AMD, ranged from 423 ngs to 2696 ngs per sample at 24 h post-injection. Even if a fraction of the tissue-associated bevasiranib enters the cell, it is likely to be effective in suppressing VEGF production. These and previously published data from our laboratory collectively provide strong evidence that after an intravitreal or subretinal injection bevasiranib does distribute to the retina and RPE-Bruchs membrane- choroid complex and therefore should effect the RNA interference mechanism resulting in gene silencing to halt the production of VEGF-A in those tissues.
